# Suboptimal Achievement of Guideline-Recommended LDL-C Targets in Older Patients Undergoing Comprehensive Geriatric Care

**DOI:** 10.3390/jcm15135066

**Published:** 2026-06-29

**Authors:** Ivan Fleisher, Karel Kostev, Dirk Bandorski, Ali Hammed, Liyibeth Florez Contreras, Christian Tanislav

**Affiliations:** 1 Department of Geriatrics, Diakonie Hospital Jung Stilling Siegen, Wichernstrasse 40, 57074 Siegen, Germany; 2 University Clinic, Philipps-University, 35043 Marburg, Germany; 3 Faculty of Medicine, Justus Liebig University, 35390 Giessen, Germany; 4 Neurological Clinic Bad Salzhausen, 63667 Nidda, Germany

**Keywords:** cholesterol-lowering therapy, older patients, LDL-C, cardiovascular risk

## Abstract

**Background:** Lowering low-density lipoprotein cholesterol (LDL-C) effectively reduces the risk of cardiovascular events. Therefore, we investigated LDL-C levels in geriatric patients undergoing comprehensive inpatient geriatric care. **Methods:** Patients aged ≥65 years who underwent inpatient comprehensive geriatric care were analyzed. Baseline, clinical, laboratory, and medical data were obtained from case records. For cardiovascular risk stratification, SCORE2, SCORE2-OP, or SMART2 was applied, and LDL-C targets for primary or secondary prevention of atherosclerotic cardiovascular disease (ASCVD) were defined. Factors associated with LDL-C values within guideline-recommended targets in the univariate analysis were entered into a logistic regression model to identify independent predictors. **Results:** Of 486 patients, 433 (median age 84.0 years; 67.2% female) were included in the final analysis. The majority of patients (371/433; 85.7%) had a very high cardiovascular risk profile. Lipid-lowering therapy (LLT) was identified in 222 patients (51.3%), while 205 patients (47.3%) had received LLT for ≥3 months. In 219 patients (98.7%), LLT was statin-based, either as monotherapy or in combination. The median LDL-C level in the entire cohort was 85 mg/dL (IQR: 63–114 mg/dL), whereas patients receiving LLT had a median LDL-C level of 66 mg/dL (IQR: 52–83 mg/dL). Overall, 193 patients (44.6%) achieved guideline-recommended LDL-C targets; among patients receiving LLT, 61.5% (126/205) were within target range. Intake of ≥5 medications per day was associated with pre-existing LLT (odds ratio: 3.036; 95% CI: 1.081–8.523; *p* = 0.035). Statin-based LLT was independently associated with achieving LDL-C targets (odds ratio: 3.383; 95% CI: 2.248–5.092; *p* < 0.001). **Conclusions:** Most patients did not achieve guideline-recommended LDL-C targets, while only half received lipid-lowering therapy, predominantly statin-based. Current risk assessment tools and approaches to polypharmacy may require adaptation for geriatric patients. Nevertheless, even the simple implementation of statin therapy alone could substantially improve cardiovascular preventive care in a large proportion of untreated older patients.

## 1. Background

In recent years, accumulating evidence has highlighted the pivotal role of lowering low-density lipoprotein cholesterol (LDL-C) in effectively reducing the risk of vascular events [1,2]. Concurrently, considerable efforts have been dedicated to the development of novel LDL-lowering therapies, culminating in the design of new agents that directly target LDL synthesis, such as small-interfering ribonucleic acids and direct PCSK9 (proprotein convertase subtilisin/kexin type 9) inhibitors [3,4,5,6,7]. However, it remains challenging for clinicians to accurately assess an individual patient’s cardiovascular risk and consequently determine appropriate LDL-C target levels to guide optimal therapeutic management [8]. To address this, different scores were developed to assess the cardiovascular risk in the clinical routine, such as SCORE2 (Systematic Coronary Risk Estimation 2), SCORE2-OP (Systematic Coronary Risk Estimation 2 Old Patients) for older adults aged ≥70 years, and the SMART2 (Secondary Manifestations of Arterial Disease 2) for those with established atherosclerotic cardiovascular disease (ASCVD) [9]. To account for additional comorbidities, such as diabetes mellitus or chronic kidney disease, further specific algorithms have been developed [9].

Lipid-lowering therapy remains beneficial even in older patients, as statins reduce the risk of myocardial infarction by approximately 39% and stroke by 24%, including among elderly individuals without established cardiovascular disease [10]. Notably, patients aged over 75 years with cardiovascular disease derive even greater absolute benefit from intensified lipid lowering, experiencing a substantially larger reduction in major cardiovascular events compared to younger patients [11]. However, treatment decisions in older adults warrant careful consideration, as deprescribing may represent an appropriate strategy in this population, since reducing medication use can be associated with fewer drug interactions and better adherence [12]. Although older patients—often recognized as a high-risk group—derive at least comparable benefit from cholesterol-lowering therapy as compared to younger individuals, recent real-world data analyses suggest uncertainty in the implementation of therapy among patients who, according to current guideline recommendations, would qualify for treatment [9,12,13,14,15,16,17,18,19].

In this regard, the present study analyzed LDL cholesterol levels in older patients admitted for inpatient comprehensive geriatric care, evaluating these values in the context of current guideline recommendations and identifying factors potentially associated with LDL levels outside the recommended range. Our study would provide important insights into the current state of care regarding lipid-lowering therapy in this specific patient population and could help identify potential strategies to improve clinical management and care delivery.

## 2. Methods

This retrospective study included patients aged ≥65 years who underwent inpatient comprehensive geriatric treatment at the Department of Geriatrics, Diakonie Hospital Jung-Stilling, Siegen, Germany, between January 2024 and December 2024. The primary objective of the presented study was to examine whether patients admitted for comprehensive geriatric care had LDL-C levels in accordance with their cardiovascular risk classification as defined by guidelines.

Baseline, clinical, laboratory, and medical history data were obtained from the hospital’s electronic case records. Clinical and medical history data routinely and systematically collected upon hospital admission are recorded electronically and stored within the hospital documentation system. As part of this standardized procedure, information is complemented through inquiries made with general practitioners and family members. This process also ensures data completeness for billing purposes. The data used in this study include, among others, vascular risk factors, additional comorbidities, information on medication use, lifestyle habits, and results from assessments performed at the time of admission. In routine blood examinations at admission, the following parameters were analyzed in the present study: complete blood count, creatinine, eGFR, GOT, GPT, GGT, CRP, glycated hemoglobin, total cholesterol, LDL-C (directly measured), HDL-C, and triglycerides. LDL-C values from patients with evidence of systemic inflammation (C-reactive protein (CRP) ≥ 2 mg/dL) were excluded from the analysis. Patients whose risk assessment did not indicate a need for LDL-C evaluation were also excluded from the final analysis.

Overall, the following inclusion criteria were applied for patient selection in the present study: (1) patients aged ≥65 years who underwent inpatient comprehensive geriatric treatment at the Department of Geriatrics, Diakonie Hospital Jung-Stilling, Siegen, Germany, between January 2024 and December 2024; and (2) patients with an indication for LDL-C–lowering therapy according to their individual cardiovascular risk classification. Patients were excluded from the analysis if they met any of the following exclusion criteria: (1) absence of an indication for LDL-C–lowering therapy based on the individual cardiovascular risk classification; and (2) current evidence of systemic inflammation, defined as a C-reactive protein level ≥2 mg/dL.

This study analyzed exclusively retrospectively collected, anonymised data; therefore, informed consent was neither required nor obtained. Under German law, the use of anonymised electronic medical data for research purposes is permitted provided specific conditions are fulfilled and does not require written informed consent or prior approval from a medical ethics committee. Nevertheless, the study protocol was submitted to the local ethics committee at Justus Liebig University Giessen (Germany), which approved the study without restrictions (protocol No AZ 111/25; approval date: 8 August 2025). All procedures were conducted in accordance with the principles of the Declaration of Helsinki (1975, revised 2013).

### 2.1. Comprehensive Geriatric Care

Patients treated in our geriatric department were referred from different hospital departments, as well as older patients referred by general practitioners and other hospitals in the region. They presented with an ongoing need for medical treatment and limitations in daily living competence, but with a positive prognosis for improvement. For this purpose, a systematic assessment is routinely performed for selecting patients for comprehensive geriatric care. Comprehensive geriatric care is therefore defined as a multi-component intervention addressing multiple health domains to develop a person-centered therapeutic plan satisfying acute medical requirements and rehabilitation needs [20].

Different members of the multidisciplinary team were involved: physicians, nursing staff, therapists, and social service workers. Functional and cognitive status were assessed and standardized at admission by physiotherapists and occupational therapists using established geriatric assessments: the Barthel index, Tinetti Score, geriatric depression scale, timed up and go test, and Mini-Mental State Examination [20].

### 2.2. Patients Stratification According to Vascular Burden

According to established guidelines, stratification of patients into primary or secondary prevention for ASCVD is required to calculate the 10-year risk of a vascular event and to determine corresponding therapeutic LDL-C targets [9]. The diagnosis of ASCVD was documented in line with the guideline if one of the following conditions was met: history of myocardial infarction or acute coronary syndrome; prior coronary or other revascularization procedures, stroke or TIA, known aortic aneurysm, known peripheral artery disease, or evidence of atherosclerotic stenosis >50% proved on coronary angiography, carotid ultrasound, or CT angiography [9]. For patients without an established diagnosis of ASCVD and thus in a primary prevention setting, three additional patient categories in line with the guidelines were defined and considered in the present analysis:

- Patients with documented chronic kidney disease and no AVCVD;
- Patients with documented diabetes mellitus and no AVCVD;
- Apparently healthy patients (no ASCVD, no diabetes mellitus, or no chronic kidney disease).

**Figure 1:**
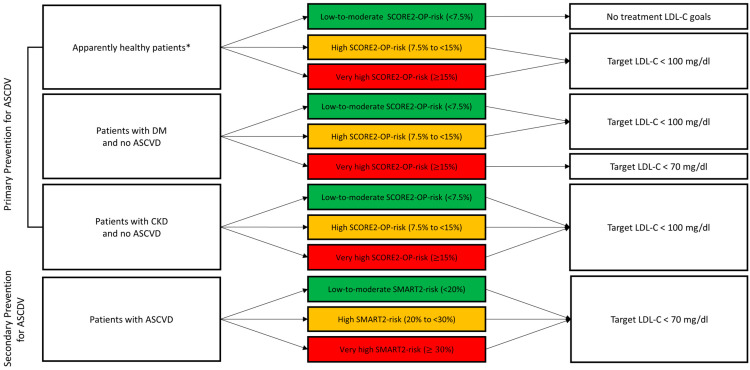
Identification of LDL-C targets as indicated by guideline STEP 1 (adapted according to [9]). The first step (STEP 1) is mandatory and includes general recommendations (such as smoking cessation and lifestyle optimization), together with specified LDL-C target values for guiding lipid-lowering therapy [9]. * refers to patients without any of ASCVD, diabetes mellitus, or chronic kidney disease. ASCVD = atherosclerotic cardiovascular disease; DM = diabetes mellitus; CKD = chronic kidney disease; LDL-C = low-density lipoprotein cholesterol.

See here also Figure 1.

### 2.3. Vascular Risk Assessment and LDL Targets

In patients undergoing primary prevention for ASCVD, the 10-year risk of a cardiovascular event was calculated using the SCORE2-OP algorithm in those aged 70 years or older and, where appropriate, the SCORE2 algorithm in individuals younger than 70 years. After application of the score, patients were categorized into low-to-moderate (<7.5%), high (7.5% to <15%), and very high (≥15%) risk categories [9].

In patients with established ASCVD (secondary prevention), the 10-year cardiovascular risk was calculated by applying the SMART2 assessment [9]. Patients were classified into low-to-moderate (<20%), high (20% to <30%), or very high (≥30%) risk categories [9,21,22].

In the present study, LDL-C targets were defined according to established guideline recommendations, based on the estimated 10-year risk of a vascular event and whether patients were in a primary or secondary prevention setting (see Figure 1) [9]. The guideline recommends a stepwise algorithm for patient management. The first step (STEP 1) is mandatory and includes general recommendations (such as smoking cessation and lifestyle optimization), together with specified LDL-C target values for guiding lipid-lowering therapy [9]. The second step (STEP 2) outlines a more stringent regimen with intensified risk factor management and should be taken into consideration, especially in patients with high-risk profiles; its application, however, depends on various factors, including 10-year CVD risk, comorbidities, frailty status, and particularly balancing lifetime risk and treatment benefit profile [9]. The latest applies notably to geriatric patients. Given this, and considering the aim of our study—which is to assess whether patients at the beginning of comprehensive geriatric care present LDL-C levels consistent with guideline recommendations—the minimum requirement according to STEP 1 was regarded as an appropriate treatment target. Therefore, in our analysis, the LDL-C target values specified for STEP 1 were used as the reference for adequate LDL-C levels in both primary and secondary prevention of ASCVD. These targets were subsequently compared with LDL-C values measured in our patients at hospital admission.

### 2.4. Statistical Analyses

All data were anonymized and entered into an Excel database for further analysis. Group comparisons were performed across relevant clinical and laboratory parameters. Continuous variables are presented as mean ± standard deviation or as median with interquartile range, depending on data distribution. Categorical variables are reported as absolute and relative frequencies. Normality of continuous variables was assessed using the Kolmogorov–Smirnov test. For comparison of categorical variables, Fisher’s exact test or the Chi-square test was applied, depending on group size. For continuous variables with normal distribution, means were compared using a two-sided independent-samples *t*-test. For non-normally distributed continuous variables, comparisons were performed using the two-sided Mann–Whitney U test. Parameters associated with the univariate analysis were included in a stepwise logistic regression analysis based on weighting, avoidance of redundancies, and other obvious dependencies. Descriptive and inferential statistical analyses were performed using SPSS software (version 22.0; IBM Corporation, Armonk, NY, USA). Figures and graphs were prepared using PowerPoint (Microsoft Corp., Redmond, WA, USA) and Excel (Microsoft Corp., Redmond, WA, USA).

## 3. Results

**Figure 2:**
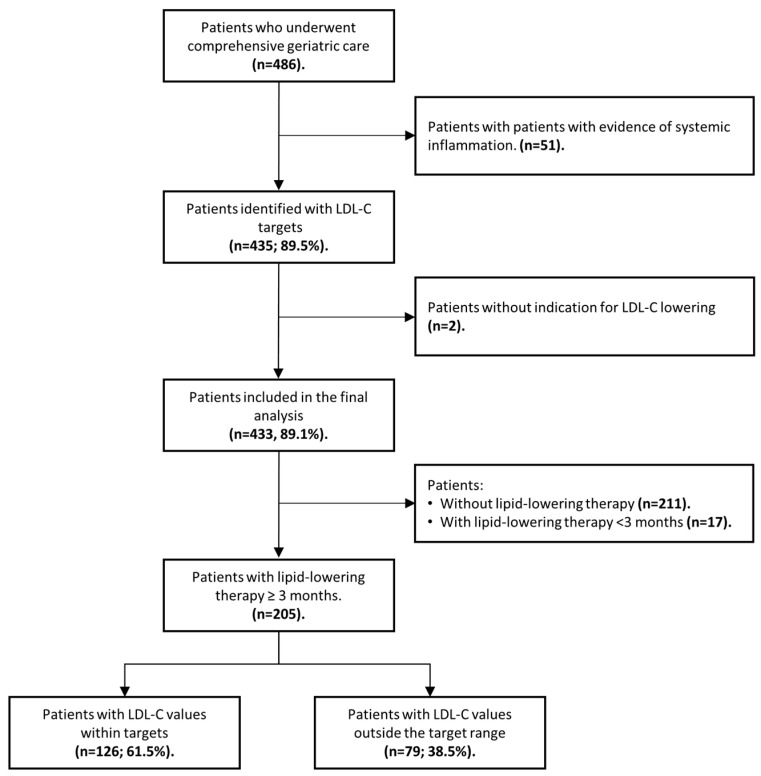
Patients treated in the geriatric department. The majority have a target for LDL-C values (433/486; 89.1%), requiring therapeutic measures. Only 205 patients had received lipid-lowering therapy for more than 3 months; among them, 126 (61.5%) had LDL-C values on admission in line with guideline recommendations.

**Figure 3:**
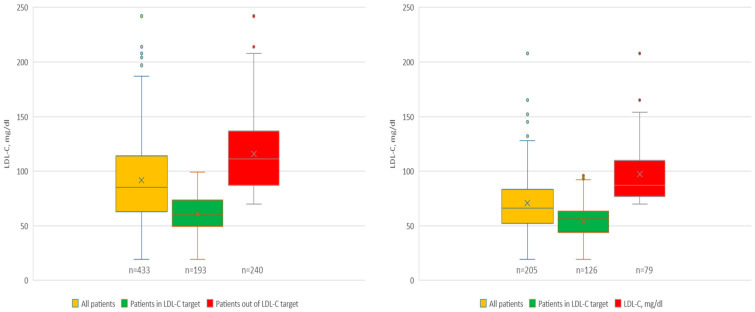
Distribution of LDL-C levels (minimum, first interquartile range, median, third interquartile range, maximum; the cross in the boxplot indicates the calculated mean value) in all patients (**left**) and those with lipid-lowering therapy over 3 months (**right**). LDL-C = low-density lipoprotein cholesterol.

All patients who underwent comprehensive geriatric care between January and December 2024 were enrolled in the study (n = 486). Patients with potential influences on lipid profiles—such as acute inflammation (defined as leucocytes >11/nl or hs-CRP >2 mg/dL) or antibiotic therapy at admission—were excluded (n = 51). According to the algorithm detailed above, an LDL-C target for therapy was identified for 433 patients (median age 84.0 years); 291 patients were female (67.2%). Out of the 433 patients included in the present study, for 193, the LDL-C values measured on admission were within the targeted range (44.6%). In 222 (45.7%) patients, a lipid-lowering therapy at admission was identified; further, 17 (3.5%) individuals underwent a lipid-lowering therapy for <3 months. Patient selection and exclusions relevant to the subsequent analysis are illustrated in Figure 2. The median LDL-C level in the entire patient group was 85 mg/dL (IQR: 63–144 mg/dL); in those patients with a lipid-lowering therapy, the median value was 66 mg/dL (IQR: 52–83 mg/dL) (Figure 3). Among the 433 patients with complete documentation, 140 patients underwent a second LDL-C measurement within 2 weeks of hospital admission. A retrospective review of the medical records revealed no explicit clinical indication for this repeated measurement. In 111 patients (79.3%), the second LDL-C measurement did not change classification with respect to being within or outside the guideline-defined LDL-C target. Among the remaining 29 patients, 14 were reclassified from outside to within the LDL-C target after the second measurement, whereas 15 moved in the opposite direction.

Patients within LDL-C target limits had lower total cholesterol, LDL-C, HDL-C, and non-HDL-C levels (*p* < 0.001), as well as lower triglyceride levels (*p* = 0.012). Patients with LDL-C levels within targets have lower values of glycated hemoglobin (median 5.5% (IQR: 5.1–6.1%) versus median 5.6% (IQR: 5.2–6.1%); *p* = 0.004) as well as lower values for blood pressure (median 123 mmHg (IQR: 115–140 mmHg) vs. median 130 mmHg (IQR: 115–145 mmHg); *p* = 0.027). Monotherapy with statins (46.9%) was the predominant management considered for LDL-C lowering. Therapy with oral antidiabetic drugs was associated with LDL-C values in the targeted range (33.2% versus 24.2%; *p* = 0.039). Patients with LDL-C values within targets had, in general, more medications and absolute intakes per day (median 14 (IQR: 11–18) versus median 13 (IQR: 9–17) intakes; *p* = 0.012).

Baseline data and additional parameters, including comorbidities, blood values, and geriatric assessment results, are summarized in Table 1; the LLT and other therapies with presentation of different agents are summarized in Table 2. Parameters that showed a clear trend in the univariate analysis were included in a stepwise logistic regression analysis. Parameter selection was based on weighting, with avoidance of redundancies and other obvious dependencies. The results of this logistic regression analysis are summarized in Table 3. For statin-based LLT (as monotherapy or in combination), the highest odds ratio for having LDL-C values within guideline targets was calculated at 3.383 (95%-CI: 2.248–5.092; *p* < 0.001). The variable ≥5 medications/day did not reach independent significance after multivariable adjustment (*p* = 0.202), likely reflecting collinearity with statin-based LLT in this multimorbid population.

When applying the second level of the guideline (STEP 2) to identify LDL-C targets in our patients, the percentage of individuals with LDL-C values within the defined targets declined from 44.6% (193/433; for STEP 1) to 32.3% (140/433). The results, taking into account LDL-C targets specified at STEP 2, are summarized in a separate Supplementary File.

**Figure 4:**
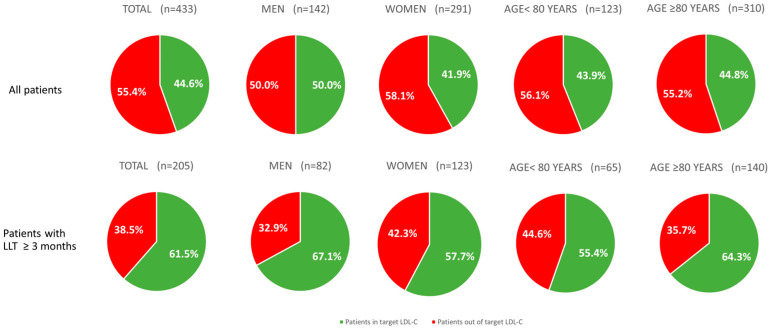
Distribution of patients in target/out of target LDL-C according to guideline STEP 1 (adapted according to [9]), according to sex and age, within all patients and patients with lipid-lowering therapy for more than 3 months. The first step (STEP 1) is mandatory and includes general recommendations (such as smoking cessation and lifestyle optimization), together with specified LDL-C target values for guiding lipid-lowering therapy [9]. LDL-C = low-density lipoprotein cholesterol; LLT = lipid-lowering therapy.

Dichotomizing the total group (n = 433) according to sex, the frequency of male versus female patients within the LDL-C values with the target remained comparable (female patients—122/291 (41.9%)—versus male patients—71/142 (50.0%); *p* = 0.112). Dichotomizing the total study group (n = 433) into patients <80 years of age and ≥80 years of age, respectively, frequencies of patients within LDL-C targets showed the same trend (patients <80 years of age: 54/123 (43.9%) versus patients ≥80 years of age: 139/310 (44.8%); *p* = 0.860). For patients who underwent lipid-lowering therapy for more than 3 months, comparable trends could be observed (see Figure 4).

**Figure 5:**
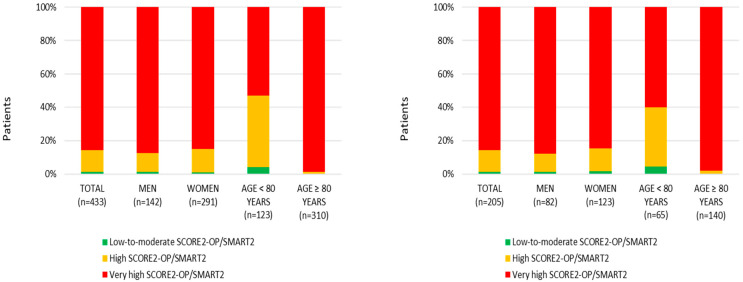
Distribution of 10-year risk of cardiovascular events within all the patients according to sex and age (**left**); distribution of 10-year risk of cardiovascular events within the patients with a lipid-lowering therapy for more than 3 months according to sex and age (**right**). SCORE2 = Systematic Coronary Risk Evaluation 2; SCORE2-OP = Systematic Coronary Risk Evaluation 2 for Older Persons; SMART2 = second SMART risk model/second version of the SMART risk score.

The distribution according to the SCORE2-OP/SMART2 categories revealed a great majority of patients with a very high risk for CVD (371/433; 85.7%); the dichotomisation into male and female patients indicated the same trend (see Figure 5). In contrast, the subgroup of patients <80 years of age, only 52.8% (65/123), presented with a very high risk for a CVD according to the SCORE2-OP/SMART2 assessment (see Figure 5). Comparable trends regarding the distribution of risk categories were observed within the subgroup of patients with a lipid-lowering therapy; these results are presented in Figure 5.

**Figure 6:**
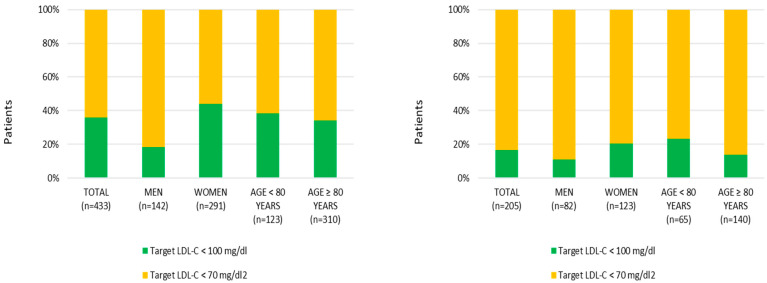
Distribution of target LDL-C within all the patients according to sex and age (**left**); distribution of target LDL-C within the patients with a lipid-lowering therapy for more than 3 months according to sex and age (**right**). LDL-C target values were determined in each patient according to the guideline (STEP 1) [9]. The first step (STEP 1) is mandatory and includes general recommendations (such as smoking cessation and lifestyle optimization), together with specified LDL-C target values for guiding lipid-lowering therapy [9]. LDL-C = low-density lipoprotein cholesterol.

In 64.7% of the study group (n = 433), a target LDL-C <70 mg/dL was determined. Dichotomizing by sex, male patients had more often an LDL-C target below 70 mg/dL than female patients (male patients—116/142, 81.7%—versus female patients—163/291, 56%; *p* < 0.001). Dichotomizing the entire group into patients <80 years versus ≥80 years of age, no clear trends could be observed (see Figure 6). This also applies when investigating the subgroup of patients with an LLT (n = 205) (see Figure 6).

Out of 433 patients with defined LDL-C targets, 205 (53.8%) had documented lipid-lowering therapy for ≥3 months. This subgroup had a median age of 83.0 years (IQR 78.0–87.0); 123 patients (60.0%) were women. Within this group, 126 patients (61.5%) had LDL-C values within the targeted range. The comparison of patients within LDL-C target values versus out of target within the subgroup of patients with an LLT (n = 205) is summarized in Table 4.

To identify factors associated with pre-existing LLT, we compared patients with and without LLT in the overall cohort (n = 433). The factor of secondary prevention was associated with a pre-existing LLT (84/228, 36.8%, versus 150/205, 73.2%; *p* < 0.001) as well as vascular risk factors such as intake of more than five medications per day, cardiovascular disease, diabetes mellitus, intake of gliflozins, alcohol abuse, or previous stroke. Results are summarized in Table 5. In the logistical regression analysis, the factor intake of ≥5 medications per day was associated with a pre-existing LLT in our patient group (odds ratio: 3.036; 95%-CI: 1.081–8.523; *p* = 0.035). The results of the logistical regression analysis are summarized in Table 6.

To assess the potential effect of sex, we analyzed the distribution of primary versus secondary ASCVD prevention and risk classification in the overall cohort. The proportion of patients in secondary prevention was higher in men than in women (104/142, 73.2%, versus 130/291, 44.7%; *p* < 0.001). No clear differences were observed in the distribution of risk categories between female and male patients. The Results are summarized in Table 7.

## 4. Discussion

Among our geriatric patients, 99.3% (433/435) had a vascular risk profile warranting intervention for LDL-C–lowering. More than 80% of these patients (n = 433) were classified as being at very high risk for experiencing a cardiovascular event. Consequently, a stringent LDL-C target of <70 mg/dL had to be applied to the majority of these individuals. However, less than half of the patients (47.3%) received lipid-lowering therapy. In our study, the intake of ≥5 medications per day emerged as a key determinant of LLT use. Statin-based therapy (as monotherapy or in combination) was the predominant form of LLT (219/222, 98.7%). Furthermore, our study unveiled in this specific group of older geriatric patients the discrepancy that, despite a general low level of measured LDL-values (median 85 mg/dL; IQR: 63–114 mg/dL), the vast majority (55.4%) exceeded guideline-determined LDL-C targets. Statin-based LLT (as monotherapy or in combination) and therapy with gliflozins had a substantial impact on whether LDL-C levels were within guideline-recommended ranges. Among the subgroup of patients with pre-existing lipid-lowering therapy (n = 205), 38.5% of the individuals remained above the guideline LDL-C goal.

In this specific group of geriatric patients, the SCORE2/SCORE2-OP/SMART2 assessment classified the vast majority (371/433; 85.7%) in the very high-risk category, thus determining for many (64.2%) the lowest LDL-C target (<70 mg/dL). In this context, it is important to note that LDL-C targets in our study were defined at the level of STEP 1 of the guideline; with treatment intensification—particularly in patients in secondary prevention—targets would be further tightened, likely resulting in even more pronounced findings.

One possible explanation for the observed underuse of lipid-lowering therapy despite the high cardiovascular risk burden may be therapeutic inertia. In routine clinical practice, physicians may hesitate to initiate or intensify lipid-lowering therapy in very old adults because of concerns regarding tolerability, polypharmacy, limited life expectancy, or uncertain benefit in frail individuals. However, accumulating evidence suggests that older patients derive substantial cardiovascular benefit from LDL-C reduction comparable to younger populations [1,2,14,15,16,17].

Another relevant aspect is the so-called frailty-treatment paradox. Patients with the highest cardiovascular risk and greatest burden of comorbidities are often less likely to receive preventive therapies, despite potentially deriving considerable benefit from treatment. In geriatric medicine, treatment decisions are frequently influenced by functional impairment, cognitive decline, and competing medical priorities, which may contribute to undertreatment of cardiovascular risk factors.

In our study, two factors were associated with LDL-C levels within target: pre-existing statin-based lipid-lowering therapy (as monotherapy or in combination) and a therapy with gliflozins. This finding also highlights that, despite the availability of newer agents, simple statin therapy can already provide substantial benefit [3,4,5,6,7]. Our study unmasked a large proportion of patients who, despite having a clear indication for LLT, did not receive even basic statin therapy. It is therefore reasonable to assume that wider use of these simple treatments alone could substantially improve the dramatic gaps in care demonstrated in our study. In our study, therapy with gliflozins was associated with lower LDL-C levels, although these substances are not primarily considered LDL-C–lowering agents [23]. This association is likely explained by the clustering of intensive cardiovascular risk management in patients receiving gliflozins rather than by a direct lipid-lowering effect of these drugs [24]. As previously reported, patients receiving gliflozins are frequently managed within structured care pathways associated with more intensive cardiovascular risk factor control and greater adherence to guideline-directed therapies [25]. Accordingly, the higher rate of LDL-C target attainment is not unexpected. An LDL-C–lowering effect of gliflozins has not been demonstrated [26]. The cardiovascular benefits of this drug class are thought to be mediated predominantly through haemodynamic, metabolic, and renoprotective mechanisms, indicating that improved LDL-C control is more likely attributable to concomitant lipid-lowering therapy and comprehensive cardiovascular care than to a direct pharmacological effect [27].

Despite the increasing availability of newer lipid-lowering agents, such as PCSK9 inhibitors and small-interfering RNA therapies, their use in our cohort was almost absent. This likely reflects limited implementation of newer therapies in routine geriatric care, possibly due to restricted access, reimbursement issues, lack of specialist involvement, or uncertainty regarding their role in frail older populations.

In our study, intake of ≥5 medications per day emerged as an important determinant of pre-existing lipid-lowering therapy. This finding raises the question of whether this result reflects a specific influence on LLT use or whether it simply indicates that, in general, more medications are required to pursue therapeutic goals. In multimorbid patients, this need for multiple drugs can be particularly challenging, especially in the context of the ongoing debate on polypharmacy and deprescribing in older patients [28]. For clinicians, this represents a difficult balance between the need to treat individual conditions and the risk that increasing the number of prescribed medications may undermine treatment effectiveness through poor adherence [29].

At this point, it is critical to discuss whether the recommended scale assessment scheme is adequate for defining therapeutic goals in clinical practice for older patients. Increasing age represents one of the main factors leading to classification into the high-risk group. However, important other factors that should guide therapeutic decision-making in older individuals—such as life expectancy at this stage of life—are not adequately considered. Therefore, it would be of benefit, particularly for the group of older geriatric patients, to consider some other factors for risk stratification and for setting therapeutic targets.

In analogy to other diseases, less stringent target values have already been defined for older patients with comorbidities, for example, for those with diabetes mellitus [30] or arterial hypertension [31]. In our study, this may partially account for the high proportion of patients who fail to have guideline-recommended LDL-C levels, irrespective of whether lipid-lowering therapy was administered. Nevertheless, it should be noted that the LDL-C values observed in our patients—except for a limited number of outliers—are generally in the lower range; yet, they still exceed the target thresholds defined by current guidelines.

In our study, functional deficits, as determined by structured functional assessments at admission, had no influence on whether patients’ LDL-C levels were within the targeted range. This finding applied to both the overall study group and the subgroup of patients who were already receiving lipid-lowering therapy. The functional outcome assessed in our study also had no impact on whether LLT was pre-existing. This finding is surprising and not directly expected; functional deterioration interferes with health awareness and adherence to therapies [32,33]. However, according to our findings, these factors do not appear to play a relevant role in LDL-C levels in older patients.

Additional factors were identified in our study associated with LDL-C levels within target, including lower HbA1c values and lower blood pressure. This may reflect a general effect whereby patients who are adequately treated for their vascular risk show improvements across multiple parameters. In case of higher HbA1c values, it could be speculated that LDL-C levels may fall outside target ranges, as this condition is pathophysiologically associated with adverse effects on lipid metabolism [34].

The high prevalence of LDL-C levels outside the target range may be in part explained by risk stratification with its stringent LDL-C targets and various external factors; however, it may also reflect the complexity and challenges of managing severely ill, multimorbid older patients [35]. Overall, taking into account possible explanations, our results indicate insufficient management of older patients in terms of achieving LDL-C target levels. Even though our findings show that pre-existing lipid-lowering therapy only modestly increased the proportion of patients achieving LDL-C targets (by 16.9%), the implementation and actual treatment of patients who require therapy remain one of the most important measures available to the treating physicians. In this context, awareness among physicians also needs to be further increased regarding the effectiveness of LDL-C lowering in reducing the risk of cardiovascular events. As demonstrated by current studies, intensive LDL-C reduction is associated with a 28% reduction in major vascular events per 1 mmol/L decrease in LDL-C compared with less intensive therapy [36], and this benefit also applies to elderly patients [9,14,15,16,17,18]. Furthermore, the observed beneficial effect of pre-existing lipid-lowering therapy, both as monotherapy and dual therapy, on lipid metabolism in our study is consistent with the evidence [37] and underscores the reliability of our data. Nevertheless, this contrasts substantially with the principle of polypharmacy in older adults and warrants critical discussion regarding the extent to which deprescribing may counteract efforts to attain LDL-C targets.

## 5. Limitations/Study Strengths

This study has several limitations. The wide confidence interval observed for secondary prevention in the logistic regression model predicting pre-existing LLT reflects reduced statistical precision due to low event counts in the reference subgroup; this estimate should be interpreted alongside the descriptive data, which robustly demonstrate the clinical association. The sample size was limited and restricted to geriatric inpatients; thus, generalizability to other patients is limited. Nevertheless, our findings provide important insights into the management of the risk factor hypercholesterolemia in older geriatric patients in routine clinical care. Although our study included only a small subset of patients within this age group, the question arises as to the extent to which our results are generalizable and reflect real-world clinical practice. Even if this assumption were valid only in part, this is of considerable concern, as it would imply that hypercholesterolemia, a major cardiovascular risk factor, is insufficiently addressed or not addressed at all in a substantial proportion of affected individuals. Another potential limitation is the possibly inadequate risk classification in this specific population of older, multimorbid patients. In light of the robustness of our findings, risk stratification tools may need to be adapted for this patient group, which would require additional clinical investigations. Future improvements in diagnostic methods will be highly important; early studies using artificial intelligence have shown promising results, with substantially better detection of pathological findings and risk stratification [38].

Our analysis is based on a selected patient group, and this should be considered when addressing the generalisability of our findings. In particular, the external validity may be influenced by local outpatient care structures, which substantially shape medical management. Previous hospitalisations may also have affected LDL-C management. Furthermore, the study region is predominantly rural, centered around a medium-sized urban hub. Despite these considerations, our findings provide a consistent picture of lipid-lowering therapy and guideline implementation in real-world clinical practice. Further studies in comparable settings are warranted to corroborate and extend these observations.

Only LDL-C values based on a single measurement at admission were included in the analysis. To address this issue, we analyzed a subgroup of 140 patients in whom a second LDL-C measurement was obtained within 2 weeks and assessed whether the second measurement changed classification. In 80% of these patients, the second LDL-C measurement did not alter classification as being within or outside the guideline-recommended target; among the remaining 20%, changes in classification were balanced between the two directions. Although the analysis of this subgroup cannot be generalized to the overall group, the measurement of variability within this subgroup indicates that this does not appear to have a substantial impact on the overall interpretation. Further studies are required in which this aspect is rigorously controlled, and LDL-C measurements are standardized and consistently assessed. To reduce bias from acute infection-related changes in LDL-C, we excluded patients with laboratory evidence of inflammation from the analysis. Interpreting a single LDL-C measurement as being at or out of target can be controversial, particularly in patients with further acute pathologies or exacerbations of chronic diseases. In this retrospective study, it was not possible to collect additional data such as adherence to therapy, intolerance to lipid-lowering therapy, dietary changes, adverse effects, contraindications, or patient preferences.

In addition, frailty was not quantified using a dedicated frailty index, although structured geriatric assessments were routinely performed. Follow-up data on cardiovascular outcomes after discharge were not available and could not be assessed.

## 6. Conclusions

In our study, it was remarkably demonstrated that in the specific group of geriatric patients, with or without lipid-lowering therapy, the measured LDL-C levels were low; yet, most patients did not have values within the limits defined by guidelines. This urges the discussion of the utility of established risk-stratification tests for older patients, while, on the other hand, it must be critically discussed to what extent the very strict LDL-C thresholds for older geriatric patients can and should be applied as they are. One of the most evident, unequivocal effects, though, is the absence of any treatment for lowering blood cholesterol; half of the patients in our study group (228/433; 52.7%) did not receive any therapy. Here, the simple use of statins alone could substantially improve the quality of care and lead to a marked reduction in the risk of vascular events in a substantial proportion of patients. Polypharmacy needs to be critically discussed in the context of guideline implementation and attainment of recommended LDL-C targets, as our findings suggest that deprescribing could have the opposite effect.

Based on the findings of the present study, future research efforts should primarily aim to simplify the transfer from risk stratification to practical treatment recommendations, with regard to individual LDL-C targets and, consequently, the initiation or intensification of lipid-lowering therapy. In the context of multimorbid geriatric patients in particular, the entire process should be simplified, and realistic and meaningful LDL-C targets should be defined in accordance with the individual patient’s situation. Until geriatric guidelines are further refined, physicians caring for older patients should adhere to existing recommendations while also considering the specific needs of older patients.

## Figures and Tables

**Table 1:** Comparison of patients with an LDL-C target with LDL-C values within versus out of target (baseline data, assessment data, comorbidities, blood values).

	Total Group (n = 433)	Patients Within LDL-C Targets (n = 193; 44.6%)	Patients out of LDL-C Targets (n = 240; 55.4%)	*p*
**Age** (median, IQR; years)	84 (79–88)	83 (78–87)	84 (79–88)	0.190
Age ≥75 years	377 (87.1%)	166 (86.0%)	211 (87.9%)	0.557
Age ≥80 years	310 (71.6%)	139 (72.0%)	171 (71.3%)	0.860
**Sex**				
Female	291(67.2%)	122 (63.2%)	169 (70.4%)	0.112
Male	142 (32.8%)	71 (36.8%)	71 (29.6%)	
**Type of prevention for ASCVD**				
Primary prevention	199(46.0%)	89 (46.1%)	110 (45.8%)	0.954
Secondary prevention	234 (54.0%)	104 (53.9%)	130 (54.2%)	
**SCORE2-OP (10-year risk for a CVD event)**				
Low-to-moderate risk for a CVD event (<7.5%)	1 (0.2%)	1 (0.5%)	0 (0.0%)	0.446
High risk for a CVD event (7.5–<15%)	41 (9.5%)	23 (11.9%)	18 (7.5%)	0.119
Very high risk for a CVD event (≥15%)	157 (36.3%)	65 (33.7%)	92 (38.3%)	0.317
SCORE2-OP (median/IQR; %)	25 (16–33)	22 (13–31)	26 (18–35)	**0.049**
**SMART2 (10-year risk for a CVD event)**				
Low-to-moderate risk for a CVD event (<20%)	4 (0.9%)	2 (1.0%)	2 (0.8%)	>0.999
High risk for a CVD event (20–<30%)	16 (3.7%)	9 (4.7%)	7 (2.9%)	0.338
Very high risk for a CVD event (≥30%)	214 (49.4%)	93 (48.2%)	121 (50.5%)	0.645
SMART2 (median/IQR; %)	68 (44–84)	63 (43–81)	73 (49–87)	0.071
**Risk related Co-morbidities**				
Cardiovascular disease	230 (53.1%)	102 (52.8%)	128 (53.3%)	0.140
Chronic kidney disease	118 (27.3%)	58 (30.1%)	60 (25.0%)	0.241
Diabetes mellitus	127 (29.3%)	58 (30.1%)	69 (28.8%)	0.767
Hypertension	385 (88.9%)	175 (90.7%)	210 (87.5%)	0.296
Systolic blood pressure (median/IQR; mmHg)	130 (115–140)	123 (115–140)	130 (115–145)	**0.027**
Heart failure	188 (43.4%)	87 (45.1%)	101 (42.1%)	0.532
Coronary heart disease	144 (33.3%)	70 (36.3%)	74 (30.8%)	0.245
Peripheral artery disease	32 (7.4%)	10 (5.2%)	22 (9.2%)	0.115
Atrial fibrillation	160 (37.0%)	77 (39.9%)	83 (34.6%)	0.255
Previous stroke	115 (26.6%)	43 (22.3%)	72 (30.0%)	0.071
Obesity	55 (12.7%)	23 (11.9%)	32 (13.3%)	0.660
BMI (median, IQR; cm/q.m.)	24.4 (21.6–27.9)	24.2 (21–27.5)	24.5 (22.1–28.1)	0.192
Abdominal circumference (median, IQR; cm)	97 (88–105)	97 (88–105)	96 (88–105)	0.669
Active smoking	35 (8.1%)	13 (6.7%)	22 (9.2%)	0.356
History of smoking	173 (40.0%)	87 (45.1%)	86 (35.8%)	0.051
**LDL-C targets**				
LDL-C <100 mg/dL	153 (35.3%)	76 (39.4%)	77 (32.1%)	0.114
LDL-C <70 mg/dL	280 (64.7%)	117 (60.6%)	163 (67.9%)	
**Blood assessment on admission**				
Total cholesterol (median/IQR; mg/dL)	155 (130–191)	129 (114–146)	184 (157–214)	**<0.001**
LDL-C (median/IQR; mg/dL)	85 (63–114)	60 (50–73)	111 (87–136)	**<0.001**
LDL-C < 100 mg/dL	278 (64.2%)	193 (100%)	85 (35.4%)	**<0.001**
HDL-C (median/IQR; mg/dL)	47 (37–58)	44 (36–52)	49 (39–52)	**<0.001**
Non-HDL-C (median/IQR; mg/dL)	108 (85–136)	82 (68–99)	132 (111–161)	**<0.001**
Triglyceride (median/IQR; mg/dL)	128 (96–165)	119 (86–159)	135 (106–167)	**0.012**
Creatinine (median/IQR; mg/dL)	0.97 (0.75–1.29)	0.99 (0.74–1.47)	0.94 (0.76–1.21)	0.135
eGFR (median/IQR; mL/min/1.73 m^2^ KOF)	66 (46–86)	62 (44–87)	68 (50–86)	0.340
Fasting glucose (median/IQR; mg/dL)	104 (93–118)	106 (93–124)	103 (93–116)	0.136
HbA1c (median/IQR; %)	5.5 (5.2–6.1)	5.5 (5.1–6.1)	5.6 (5.2–6.1)	**0.004**
GGT (median/IQR; U/L)	40 (21–83)	41 (23–89)	39 (21–79)	0.403
GOT (median/IQR; U/L)	27 (22–37)	29 (22–41)	27 (21–35)	0.053
GPT (median/IQR; U/L)	20 (14–30)	20 (14–32)	19 (14–28)	0.252
**Co-morbidities**				
Dementia	75 (17.3%)	29 (15.0%)	46 (19.2%)	0.258
Polyneuropathy	37 (8.5%)	17 (8.8%)	20 (8.3%)	0.861
Depression	102 (23.6%)	40 (20.7%)	62 (25.8%)	0.213
Chronic obstructive pulmonary disease	51 (11.8%)	24 (12.4%)	27 (11.3%)	0.704
Liver disease	15 (3.5%)	9 (4.7%)	6 (2.5%)	0.221
Hypothyroidism	156 (36.0%)	62 (32.1%)	94 (39.2%)	0.129
Hyperthyroidism	6 (1.4%)	4 (2.1%)	2 (0.8%)	0.414
Alcohol abuse	31 (7.2%)	32 (16.6%)	26 (10.8%)	0.081
**Functional assessment at admission (all patients)**				
Barthel index (median, IQR)	45 (35–60)	45 (35–60)	47 (35–60)	0.361
Tinneti geriatric assessment (median, IQR)	12 (5–18)	12 (4–18)	12 (5–18)	0.589
Geriatric depression scale (median, IQR)	4 (2–6)	3 (2–6)	4 (2–6)	0.663
Geriatric depression scale >5	115 (26.6%)	50 (25.9%)	65 (27.1%)	0.783
Timed up and go (median, IQR)	4 (3–5)	4 (3–5)	4 (3–5)	0.174
MMSE (median, IQR)	26 (22–28)	26 (22–28)	26 (21–28)	0.641

LDL-C = Low-Density-Lipoprotein Cholesterol.; IQR = Interquartile Range; ASCVD = Atherosclerotic Cardiovascular Disease; CVD = Cardiovascular Disease; SCORE2 = Systematic Coronary Risk Evaluation 2; SCORE2-OP = Systematic Coronary Risk Evaluation 2 for Older Persons; SMART2 = second SMART risk model/second version of the SMART risk score; eGFR = estimated glomerular filtration rate; IQR = interquartile range; KOF = body surface area; HbA1c = glycated hemoglobin; GGT = gamma-glutamyl transferase; GOT = glutamate oxaloacetate transaminase; GPT = glutamate pyruvate transaminase; BMI = body mass index; MMSE = Mini-Mental State Examination.

**Table 2:** Comparison of patients with an LDL-C target with LDL-C values within versus out of target (lipid-lowering therapy, other medication).

	Total Group (n = 433)	Patients Within LDL-C Targets (n = 193; 44.6%)	Patients out of LDL-C Targets (n = 240; 55.4%)	*p*
**Lipid-lowering therapy**				
Lipid-lowering therapy of any duration	222 (51.3%)	131 (67.9%)	91 (37.9%)	**<0.001**
<3 months	205 (47.3%)	5 (2.6%)	12 (5.0%)	0.199
≥3 months	17 (3.9%)	126 (65.3%)	79 (32.9%)	**<0.001**
Statine monotherapy	203 (46.9%)	118 (61.1%)	85 (35.4%)	**<0.001**
Statin-based LLT ^¥^	219 (50.6%)	131 (67.9%)	88 (36.7%)	**<0.001**
Ezetimib monotherapy	3 (0.7%)	0 (0.0%)	3 (1.3%)	0.257
Statine with ezetimib	14 (3.2%)	11 (5.7%)	3 (1.3%)	**0.012**
Statine with ezetimib and a fibrate	1 (0.2%)	1 (0.5%)	0 (0.0%)	0.446
Statine with ezetimib and bempedoic acid	1 (0.2%)	1 (0.5%)	0 (0.0%)	0.446
**Statins (distribution of substances and doses)**				
Atorvastatin 10 mg	19 (4.4%)	14 (7.3%)	5 (2.1%)	**0.009**
Atorvastatin 20 mg	54 (12.5%)	27 (14.0%)	27 (11.3%)	0.391
Atorvastatin 30 mg	1 (0.2%)	0 (0.0%)	1 (0.4%)	>0.999
Atorvastatin 40 mg	27 (6.2%)	17 (8.8%)	10 (4.2%)	**0.047**
Atorvastatin 80 mg	1 (0.2%)	0 (0.0%)	1 (0.4%)	>0.999
Simvastatin 10 mg	10 (2.3%)	4 (2.1%)	6 (2.5%)	>0.999
Simvastatin 20 mg	20 (4.6%)	8 (4.1%)	12 (5.0%)	0.674
Simvastatin 30 mg	1 (0.2%)	1 (0.5%)	0 (0.0%)	0.446
Simvastatin 40 mg	18 (4.2%)	11 (5.7%)	7 (2.9%)	0.149
Rosuvastatin 5 mg	6 (1.4%)	5 (2.6%)	1 (0.4%)	0.093
Rosuvastatin 10 mg	16 (3.7%)	13 (6.7%)	3 (1.3%)	**0.004**
Rosuvastatin 20 mg	35 (8.1%)	24 (12.4%)	11 (4.6%)	**0.003**
Rosuvastatin 40 mg	5 (1.2%)	4 (2.1%)	1 (0.4%)	0.177
Pravastatin 20 mg	2 (0.5%)	0 (0.0%)	2 (0.8%)	0.505
Pravastatin 40 mg	3 (0.7%)	3 (1.6%)	0 (0.0%)	0.088
Fluvastatin 40 mg	1 (0.2%)	0 (0.0%)	1 (0.4%)	>0.999
**Therapy**				
Number of medications (median, IQR, times)	10 (8–13)	10 (8–14)	10 (7–12)	**0.001**
Number of intakes (median, IQR, times)	14 (10–17)	14 (11–18)	13 (9–17)	**0.012**
Intake of ≥5 medications per day	403 (93.1%)	186 (96.4%)	217 (90.4%)	**0.015**
ACE inhibitors	297 (68.6%)	135 (69.9%)	162 (67.5%)	0.585
Ramipril	100 (23.1%)	40 (20.7%)	60 (25.0%)	0.294
Enalapril	13 (3.0%)	6 (3.1%)	7 (2.9%)	0.907
Lisinopril	10 (2.3%)	6 (3.1%)	4 (1.7%)	0.352
Quinapril	2 (0.5%)	1 (0.5%)	1 (0.4%)	>0.999
Perindopril	2 (0.5%)	2 (1.0%)	0 (0.0%)	0.198
Benalapril	1 (0.2%)	0 (0.0%)	1 (0.4%)	>0.999
Candesartan	104 (24.0%)	48 (24.9%)	56 (23.3%)	0.710
Valsartan	46 (10.6%)	25 (13.0%)	21 (8.8%)	0.158
Telmisartan	7 (1.6%)	3 (1.6%)	4 (1.7%)	>0.999
Irbesartan	5 (1.2%)	2 (1.0%)	3 (1.3%)	>0.999
Olmesartan	3 (0.7%)	0 (0.0%)	3 (1.3%)	0.257
Losartan	4 (0.9%)	2 (1.0%)	2 (0.8%)	>0.999
Thiazide-like diuretics	77 (17.8%)	42 (21.8%)	35 (14.6%)	0.052
Systemic corticosteroids	45 (10.4%)	18 (9.3%)	27 (11.3%)	0.514
NSAIDs	73 (16.9%)	31 (16.9%)	42 (17.5%)	0.691
Ibuprofen	24 (5.5%)	10 (5.2%)	14 (5.8%)	0.768
Diclofenac	9 (2.1%)	4 (2.1%)	5 (2.1%)	>0.999
Etoricoxib	16 (3.7%)	5 (2.6%)	11 (4.6%)	0.275
Celecoxib	20 (4.6%)	9 (4.7%)	11 (4.6%)	0.969
Naproxen	4 (0.9%)	3 (1.6%)	1 (0.4%)	0.328
Antidepressants	86 (19.9%)	35 (18.1%)	51 (21.3%)	0.419
Sedatives	97 (22.4%)	41 (21.2%)	56 (23.3%)	0.604
Anticoagulants	154 (35.6%)	78 (40.4%)	76 (31.7%)	0.059
Phenprocoumon	9 (2.1%)	5 (2.6%)	4 (1.7%)	0.520
Apixaban	111 (25.6%)	58 (30.1%)	53 (22.1%)	0.059
Edoxaban	17 (3.9%)	10 (5.3%)	7 (2.9%)	0.228
Dabigatran	5 (1.2%)	2 (1.0%)	3 (1.3%)	>0.999
Rivaraxaban	12 (2.8%)	3 (1.6%)	9 (3.8%)	0.240
Antiplatelet drugs	149 (34.4%)	65 (33.7%)	84 (35.0%)	0.774
Aspirin	118 (27.3%)	51 (26.4%)	67 (27.9%)	0.729
Clopidogrel	15 (3.5%)	3 (1.6%)	12 (5.0%)	0.064
DAPT	16 (3.7%)	11 (5.7%)	5 (2.1%)	**0.047**
Oral antidiabetic drugs	122 (28.2%)	64 (33.2%)	58 (24.2%)	**0.039**
Metformin	56 (12.9%)	26 (13.5%)	30 (12.5%)	0.765
Gliptins	33 (7.6%)	15 (7.8%)	18 (7.5%)	0.916
Gliflozins	68 (15.7%)	42 (21.8%)	26 (10.8%)	**0.002**
Glimepirid	2 (0.5%)	1 (0.5%)	1 (0.4%)	>0.999
Insulin application	45 (10.4%)	21 (10.9%)	24 (10.0%)	0.765

^¥^ as monotherapy or in combination.; LDL-C = Low-Density-Lipoprotein Cholesterol; IQR = Interquartile Range; ACE = angiotensin-converting enzyme; NSAIDs = non-steroidal anti-inflammatory drugs; DAPT = Dual Antiplatelet Therapy.

**Table 3:** Comparison of patients with LDL-C values within versus out of target (n = 433); (logistic regression analysis of selected parameters for identifying factors associated with LDL-C values within targets according to the guideline).

Selected Parameters in n = 433 Patients	Odds Ratio	CI Interval	*p* *	*p* **
Statin-based LLT ^¥^; n = 219 (50.6%)	3.383	2.248–5.092	<0.001	<0.001
Intake of ≥5 medications per day; n = 403 (93.1%)	1.799	0.729–4.440	0.015	0.202
Therapy with gliflozins; n = 68 (15.7%)	1.857	1.062–3.246	0.002	0.030

LDL-C = Low-Density-Lipoprotein Cholesterol.; LLT = Lipid-Lowering Therapy; * = *p*-value calculated in the univariate analysis; ** = *p*-value calculated in the logistical regression analysis; ^¥^ as monotherapy or in combination.

**Table 4:** Comparison of patients on lipid-lowering therapy with LDL-C values within versus out of target.

	Total Group (n = 205)	Patients on LLT in LDL-C Target (n = 126; 61.5%)	Patients on LLT Out of LDL-C Target (n = 79; 38.5%)	*p*
**Age** (median, IQR; years)	83 (78–87)	83 (78–86)	83 (77–87)	0.967
Age ≥75 years	178 (86.8%)	110 (87.3%)	68 (86.1%)	0.801
Age ≥80 years	140 (68.3%)	90 (71.4%)	50 (63.3%)	0.223
**Sex**				
Female	123 (60.0%)	71 (56.3%)	52 (65.8%)	0.178
Male	82 (40.0%)	55 (43.7%)	27 (34.2%)	
**Type of prevention for ASCVD**				
Primary prevention	55 (26.8%)	33 (26.2%)	22 (27.8%)	0.794
Secondary prevention	150 (73.2%)	93 (73.8%)	57 (72.2%)	
**SCORE2-OP (10-year risk for a CVD event)**				
Low-to-moderate risk for a CVD event (<7.5%)	0 (0.0%)	0 (0.0%)	0 (0.0%)	>0.999
High risk for a CVD event (7.5–<15%)	17 (8.3%)	13 (10.3%)	4 (5.1%)	0.206
Very high risk for a CVD event (≥15%)	38 (18.5%)	20 (15.9%)	18 (22.7%)	0.215
SCORE2-OP (median/IQR; %)	21 (13–29)	19 (13–27)	26 (18–30)	0.073
**SMART2 (10-year risk for a CVD event)**				
Low-to-moderate risk for a CVD event (<20%)	3 (1.5%)	2 (1.6%)	1 (1.3%)	>0.999
High risk for a CVD event (20–<30%)	9 (4.4%)	7 (5.5%)	2 (2.5%)	0.487
Very high risk for a CVD event (≥30%)	138 (67.3%)	84 (66.7%)	54 (68.4%)	0.802
SMART2 (median/IQR; %)	63 (43–82)	63 (43–83)	62 (45–81)	0.816
**Risk related Co-morbidities**				
Cardiovascular disease	147 (71.7%)	92 (73.0%)	55 (69.6%)	0.599
Chronic kidney disease	59 (28.8%)	39 (31.0%)	20 (25.3%)	0.386
Diabetes mellitus	80 (39.0%)	48 (38.1%)	32 (40.5%)	0.731
Hypertension	192 (93.7%)	118 (93.7%)	73 (92.4%)	0.731
Systolic blood pressure (median/IQR; mmHg)	125 (115–140)	123 (115–140)	130 (118–144)	0.098
Heart failure	99 (48.3%)	58 (46.0%)	41 (51.9%)	0.413
Coronary heart disease	103 (50.2%)	66 (52.4%)	37 (46.8%)	0.440
Peripheral artery disease	19 (9.3%)	9 (7.1%)	10 (12.7%)	0.185
Atrial fibrillation	85 (41.5%)	54 (42.9%)	31 (39.2%)	0.609
Previous stroke	67 (32.7%)	38 (20.2%)	29 (36.7%)	0.331
Obesity	35 (17.1%)	22 (17.5%)	13 (16.5%)	0.852
BMI (median, IQR; cm/q.m.)	24.8 (22–28.4)	24.6 (21.3–28.2)	24.9 (22.7–28.4)	0.560
Abdominal circumference (median, IQR; cm)	98 (90–105)	98 (90–107)	97 (90–104)	0.657
Active smoking	14 (6.8%)	7 (5.6%)	7 (8.9%)	0.361
History of smoking	91 (44.4%)	62 (49.2%)	29 (36.7%)	0.080
**LDL-C targets**				
LDL-C <100 mg/dL	34 (16.6%)	24 (19.0%)	10 (12.7%)	0.231
LDL-C <70 mg/dL	171 (83.4%)	102 (81.0%)	69 (87.3%)	
**Blood assessment on admission**				
Total cholesterol (median/IQR; mg/dL)	135 (117–157)	121 (107–135)	162 (141–187)	**<0.001**
LDL-C (median/IQR; mg/dL)	66 (52–83)	56 (44–63)	87 (77–108)	**<0.001**
LDL-C < 100 mg/dL	177 (86.3%)	126 (100%)	51 (64.6%)	**<0.001**
HDL-C (median/IQR; mg/dL)	43 (36–52)	42 (35–52)	45 (36–55)	0.375
Non-HDL-C (median/IQR; mg/dL)	89 (70–109)	75 (64–87)	112 (101–135)	**<0.001**
Triglyceride (median/IQR; mg/dL)	124 (92–164)	120 (84–160)	129 (105–164)	0.056
Creatinine (median/IQR; mg/dL)	0.99 (0.79–1.36)	1.01 (0.8–1.53)	0.97 (0.79–1.21)	0.061
eGFR (median/IQR; mL/min/1.73 m^2^ KOF)	63 (45–85)	60 (42–84)	67 (51–84)	0.130
Fasting glucose (median/IQR; mg/dL)	107 (94–127)	110 (95–129)	105 (92–120)	0.267
HbA1c (median/IQR; %)	5.7 (5.3–6.4)	5.7 (5.3–6.5)	5.7 (5.4–6.3)	0.076
GGT (median/IQR; U/L)	42 (25–83)	45 (26–89)	39 (23–73)	0.270
GOT (median/IQR; U/L)	28 (22–37)	30 (23–43)	27 (21–32)	**0.019**
GPT (median/IQR; U/L)	20 (15–31)	22 (15–36)	18 (13–26)	**0.019**
**Lipid-lowering therapy**				
Statine monotherapy	186 (90.7%)	113 (89.7%)	73 (92.4%)	0.513
Ezetimib monotherapy	3 (1.5%)	0 (0.0%)	3 (3.8%)	0.056
Statine with ezetimib	14 (6.8%)	11 (8.7%)	3 (3.8%)	0.256
Statine with ezetimib and a fibrate	1 (0.5%)	1 (0.8%)	0 (0.0%)	>0.999
Statine with ezetimib and bempedoic acid	1 (0.5%)	1 (0.8%)	0 (0.0%)	>0.999
**Co-morbidities**				
Dementia	29 (14.1%)	14 (11.1%)	15 (19.0%)	0.115
Polyneuropathy	18 (8.8%)	13 (10.3%)	5 (6.3%)	0.326
Depression	52 (25.4%)	28 (22.2%)	24 (30.4%)	0.191
Abdominal circumference (median, IQR; cm)	98 (90–105)	98 (90–107)	97 (90–104)	0.657
Liver disease	9 (4.4%)	8 (6.3%)	1 (1.3%)	0.158
Hypothyroidism	73 (35.6%)	44 (34.9%)	29 (36.7%)	0.795
Hyperthyroidism	4 (2.0%)	3 (2.4%)	1 (1.3%)	>0.999
Alcohol abuse	14 (6.8%)	13 (10.3%)	1 (1.3%)	**0.011**
**Therapy**				
Number of medications (median, IQR, times)	12 (9–14)	11 (9–14)	12 (9–14)	0.933
Number of intakes (median, IQR, times)	15 (12–20)	16 (13–20)	15 (12–19)	0.342
Intake of ≥5 medications per day	200 (97.6%)	125 (99.2%)	75 (94.9%)	0.074
ACE inhibitors	152 (74.1%)	94 (74.6%)	58 (73.4%)	0.850
Thiazides/Thiazide analogs	43 (21.0%)	31 (24.6%)	12 (15.2%)	0.107
Systemic corticosteroids	23 (11.2%)	16 (12.7%)	7 (8.9%)	0.397
NSAIDs	35 (17.1%)	24 (19.0%)	11 (13.9%)	0.343
Antidepressants	48 (23.4%)	26 (20.6%)	22 (27.8%)	0.235
Sedatives	54 (26.3%)	31 (24.6%)	23 (29.1%)	0.475
Anticoagulants	88 (42.9%)	59 (46.8%)	29 (36.7%)	0.154
Antiplatelet drug	91 (44.4%)	53 (42.1%)	38 (48.1%)	0.397
Oral antidiabetic drugs	79 (38.5%)	51 (40.5%)	28 (35.4%)	0.471
Insulin	19 (9.3%)	15 (11.9%)	4 (5.1%)	0.100
**Functional assessment at admission (all patients)**				
Barthel index (median, IQR)	45 (35–60)	45 (35–60)	45 (30–60)	0.456
Tinneti geriatric assessment (median, IQR)	11 (5–18)	12 (5–18)	11 (4–17)	0.278
Geriatric depression scale (median, IQR)	4 (2–6)	4 (2–6)	4 (2–5)	0.988
Geriatric depression scale >5	51 (24.9%)	34 (27.0%)	17 (21.5%)	0.378
Timed up and go (median, IQR)	4 (3–5)	4 (3–5)	4 (3–5)	0.509
MMSE (median, IQR)	26 (22–28)	26 (22–28)	25 (21–28)	0.443

LDL-C = low-density lipoprotein cholesterol; IQR = interquartile range; ASCVD = atherosclerotic cardiovascular disease; CVD = cardiovascular disease; SCORE2 = Systematic Coronary Risk Evaluation 2; SCORE2-OP = Systematic Coronary Risk Evaluation 2 for Older Persons; SMART2 = second SMART risk model/second version of the SMART risk score; eGFR = estimated glomerular filtration rate; KOF = body surface area; HbA1c = glycated hemoglobin; GGT = gamma-glutamyl transferase; GOT = glutamate oxaloacetate transaminase; GPT = glutamate pyruvate transaminase; ACE = angiotensin-converting enzyme; NSAIDs = non-steroidal anti-inflammatory drugs; BMI = body mass index; MMSE = Mini-Mental State Examination.

**Table 5:** Comparison of patients with a pre-existing lipid-lowering therapy on admission versus those without.

	Total Group (n = 433)	Patients with LLT (n = 205; 47.3%)	Patients Without LLT (n = 228; 52.7%)	*p*
**Age** (median, IQR; years)	84 (79–88)	83 (78–87)	84 (79–88)	**0.032**
Age ≥75 years	377	178 (86.8%)	199 (87.3%)	0.889
Age ≥80 years	310	140 (68.3%)	170 (74.6%)	0.149
**Sex**				
Female	291(67.2%)	123 (60.0%)	168 (73.7%)	**0.002**
Male	142 (32.8%)	82 (40.0%)	60 (26.3%)	
**Type of prevention for ASCVD**				
Primary prevention	199(46.0%)	55 (26.8%)	144 (63.2%)	**<0.001**
Secondary prevention	234 (54.0%)	150 (73.2%)	84 (36.8%)	
**Co-morbidities**				
Cardiovascular disease	230 (53.1%)	147 (71.7%)	83 (36.4%)	**<0.001**
Chronic kidney disease	118 (27.3%)	59 (28.8%)	59 (25.9%)	0.498
Diabetes mellitus	127 (29.3%)	80 (39.0%)	47 (20.6%)	**<0.001**
Hypertension	385 (88.9%)	192 (93.7%)	194 (85.1%)	**0.007**
Heart failure	188 (43.4%)	99 (48.3%)	89 (39.0%)	**0.052**
Coronary heart disease	144 (33.3%)	103 (50.2%)	41 (18.0%)	**<0.001**
Peripheral artery disease	32 (7.4%)	19 (9.3%)	13 (5.7%)	0.157
Atrial fibrillation	160 (37.0%)	85 (41.5%)	75 (32.9%)	0.065
Previous stroke	115 (26.6%)	67 (32.7%)	48 (21.1%)	**0.006**
Dementia	75 (17.3%)	29 (14.1%)	46 (20.2%)	0.098
Polyneuropathy	37 (8.5%)	18 (8.8%)	19 (8.3%)	0.868
Depression	102 (23.6%)	52 (25.4%)	50 (21.9%)	**0.400**
Chronic obstructive pulmonary disease	51 (11.8%)	29 (14.1%)	22 (9.6%)	0.147
Obesity	55 (12.7%)	35 (17.1%)	20 (8.8%)	**0.010**
BMI (median, IQR; cm/q.m.)	24.4 (21.6–27.9)	24.8 (22–28.4)	23.9 (21.5–27.4)	**0.049**
Abdominal circumference (median, IQR; cm)	97 (88–105)	98 (90–105)	95 (86.2–105)	**0.046**
Liver disease	15 (3.5%)	9 (4.4%)	6 (2.6%)	0.318
Hypothyroidism	156 (36.0%)	73 (35.6%)	83 (36.4%)	0.864
Hyperthyroidism	6 (1.4%)	4 (2.0%)	2 (0.9%)	0.429
Alcohol abuse	31 (7.2%)	14 (6.8%)	44 (19.3%)	**<0.001**
Active smoking	35 (8.1%)	14 (6.8%)	21 (9.2%)	0.364
History of smoking	173 (40.0%)	91 (44.4%)	82 (36.0%)	0.074
**Therapy**				
Number of medications (median, IQR, times)	10 (8–13)	12 (9–14)	8 (6–11)	**<0.001**
Number of intakes (median, IQR, times)	14 (10–17)	15 (12–20)	12 (8–16)	**<0.001**
Intake of ≥5 medications per day	403 (93.1%)	200 (97.6%)	203 (89.0%)	**<0.001**
ACE inhibitors	297 (68.6%)	152 (74.1%)	145 (63.6%)	**0.018**
Thiazides/Thiazide analogs	77 (17.8%)	43 (21.0%)	34 (14.9%)	0.099
Systemic corticosteroids	45 (10.4%)	23 (11.2%)	22 (9.6%)	0.593
NSAIDs	73 (16.9%)	35 (17.1%)	38 (16.7%)	0.910
Antidepressants	86 (19.9%)	48 (23.4%)	38 (16.7%)	**0.079**
Sedatives	97 (22.4%)	54 (26.3%)	43 (18.9%)	**0.062**
Anticoagulants	154 (35.6%)	88 (42.9%)	66 (28.9%)	**0.002**
Antiplatelet drug	149 (34.4%)	91 (44.4%)	58 (25.4%)	**<0.001**
Oral antidiabetic drugs	122 (28.2%)	79 (38.5%)	43 (18.9%)	**<0.001**
Insulin	45 (10.4%)	19 (9.3%)	26 (11.4%)	0.467
**Functional assessment at admission (all patients)**				
Barthel index (median, IQR)	45 (35–60)	45 (35–60)	47.5 (35–60)	0.100
Tinneti geriatric assessment (median, IQR)	12 (5–18)	11 (5–18)	13 (5–18)	0.158
Geriatric depression scale (median, IQR)	4 (2–6)	4 (2–6)	3 (2–6)	0.267
Geriatric depression scale >5	115 (26.6%)	51 (24.9%)	64 (28.1%)	0.453
Timed up and go (median, IQR)	4 (3–5)	4 (3–5)	4 (3–5)	0.089
MMSE (median, IQR)	26 (22–28)	26 (22–28)	26 (21–29)	0.418

LDL-C = Low-Density-Lipoprotein Cholesterol; IQR = Interquartile Range; ASCVD = Atherosclerotic Cardiovascular Disease; CVD = Cardiovascular Disease; SCORE2 = Systematic Coronary Risk Evaluation 2; SCORE2-OP = Systematic Coronary Risk Evaluation 2 for Older Persons; SMART2 = second SMART risk model/second version of the SMART risk score; eGFR = estimated glomerular filtration rate; IQR = interquartile range; KOF = body surface area; HbA1c = glycated hemoglobin; GGT = gamma-glutamyl transferase; GOT = glutamate oxaloacetate transaminase; GPT = glutamate pyruvate transaminase; ACE = angiotensin-converting enzyme; NSAIDs = non-steroidal anti-inflammatory drugs; BMI = body mass index; MMSE = Mini-Mental State Examination.

**Table 6:** Comparison of patients with LLT versus without LLT (n = 433); (logistic regression analysis of selected parameters for identifying factors associated with LLT in patients admitted for comprehensive geriatric care).

Selected Parameters in n = 433 Patients	Odds Ratio	CI Interval	*p* *	*p* **
Male sex; n = 142 (32.8%)	1.351	0.856–2.130	0.002	0.196
Secondary prevention for ASCVD; n = 234 (54.0%)	6.745	0.679–67.058	<0.001	0.103
Cardiovascular disease; n = 230 (53.1%)	0.592	0.060–5.832	<0.001	0.654
Alcohol abuse; n = 31 (7.2%)	0.803	0.350–1.844	<0.001	0.606
Intake of ≥5 medications per day; n = 403 (93.1%)	3.036	1.081–8.523	<0.001	0.035

LDL-C = Low-Density-Lipoprotein Cholesterol; ASCVD = Atherosclerotic Cardiovascular Disease; * = *p*-value calculated in the univariate analysis; ** = *p*-value calculated in the logistical regression analysis.

**Table 7:** Comparison of patients with a pre-existing lipid-lowering therapy on admission versus those without.

	Total Group (n = 433)	Female Patients (n = 291; 67.2%)	Male Patients (n = 142; 32.8%)	*p*
**Age** (median, IQR; years)	84 (79–88)	84 (79–88)	83 (78–87)	0.092
**Type of prevention for ASCVD**				
Primary prevention	199(46.0%)	161 (55.3%)	38 (26.8%)	<0.001
Secondary prevention	234 (54.0%)	130 (44.7%)	104 (73.2%)	
**SCORE2-OP (10-year risk for a CVD event)**				
Low-to-moderate risk for a CVD event (<7.5%)	1 (0.2%)	0 (0.0%)	1 (0.7%)	0.191
High risk for a CVD event (7.5–<15%)	41 (9.5%)	32 (11.0%)	9 (6.3%)	0.602
Very high risk for a CVD event (≥15%)	157 (36.3%)	129 (44.3%)	28 (19.7%)	0.382
SCORE2-OP (median/IQR; %)	25 (16–33)	28 (17–34)	20 (14–28)	0.102
**SMART2 (10-year risk for a CVD event)**				
Low-to-moderate risk for a CVD event (<20%)	4 (0.9%)	3 (1.0%)	1 (0.7%)	0.631
High risk for a CVD event (20–<30%)	16 (3.7%)	9 (3.1%)	7 (4.9%)	0.954
Very high risk for a CVD event (≥30%)	214 (49.4%)	118 (40.6%)	96 (67.7%)	0.676
SMART2 (median/IQR; %)	68 (44–84)	66 (44–82)	72 (47–87)	0.089

IQR = interquartile range; ASCVD = atherosclerotic cardiovascular disease; CVD = cardiovascular disease; SCORE2 = Systematic Coronary Risk Evaluation 2; SCORE2-OP = Systematic Coronary Risk Evaluation 2 for Older Persons; SMART2 = second SMART risk model/second version of the SMART risk score.

## Data Availability

The data presented in this study are available on request from the corresponding author.

## Supplementary Materials

The following supporting information can be downloaded at https://www.mdpi.com/article/10.3390/jcm15135066/s1.
