# Supplementary material for: Suboptimal Achievement of Guideline-Recommended LDL-C Targets in Older Patients Undergoing Comprehensive Geriatric Care
Source: J Clin Med. 2026 Jun 29;15(13):5066. doi: 10.3390/jcm15135066 (PMC13363224; doi:10.3390/jcm15135066)
Supplement: Supplementary file 1 [file jcm-15-05066-s001.zip › jcm-4364056-supplementary.pdf]

Figure 1s. Identification of LDL-C targets as indicated by guideline STEP 2 (adapted according to [1]). The first step (STEP 1) is mandatory and includes general recommendations (such as smoking cessation and lifestyle optimization), together with specified LDL-C target values for guiding lipid-lowering therapy [1]. The second step (STEP 2) outlines a more stringent regimen with intensified risk factor management und should be taken into consideration especially in patients with high-risk profiles; its application, however, depends on various factors, including 10-year CVD risk, comorbidities, frailty status, and particularly balancing lifetime risk and treatment benefit profile [1].

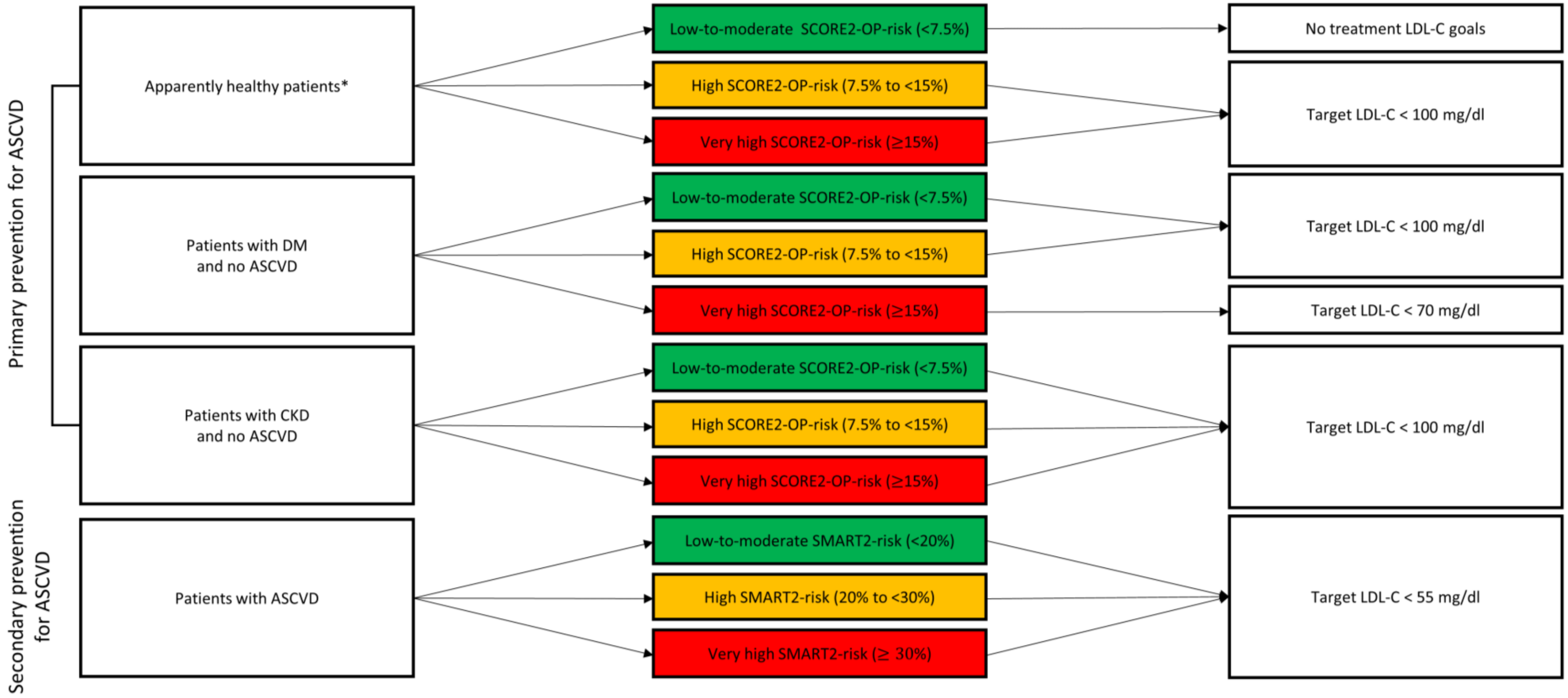

*refers to patients without any of ASCVD, diabetes mellitus or chronic kidney disease.
ASCVD = atherosclerotic cardiovascular disease, DM = diabetes mellitus; CKD = chronic kidney disease, LDL-C = low-density lipoprotein cholesterol

Figure 4s. Distribution of patients in target/out of target LDL-C according to guideline STEP 2 (adapted according to [1]) between according to sex and age within all patients and patients with lipid-lowering therapy more than 3 months. The first step (STEP 1) is mandatory and includes general recommendations (such as smoking cessation and lifestyle optimization), together with specified LDL-C target values for guiding lipid-lowering therapy [1]. The second step (STEP 2) outlines a more stringent regimen with intensified risk factor management und should be taken into consideration especially in patients with high-risk profiles; its application, however, depends on various factors, including 10-year CVD risk, comorbidities, frailty status, and particularly balancing lifetime risk and treatment benefit profile [1].

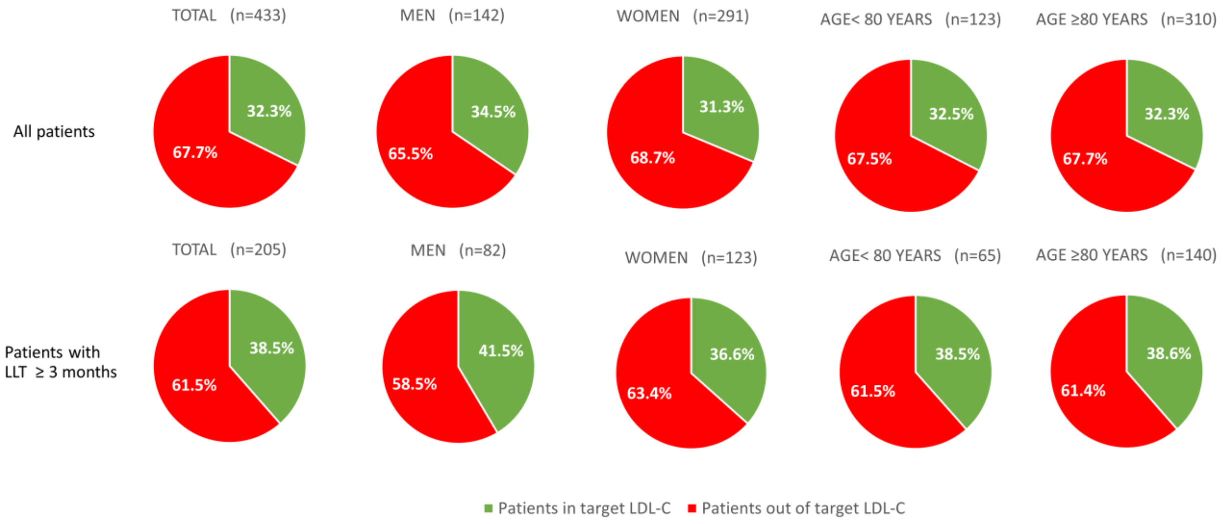

LDL-C = Low-Density-Lipoprotein Cholesterol
LLT= Lipid-Lowering Therapy

Figure 6s. Distribution of target LDL-C within all the patients according to sex and age (left); distribution of target LDL-C within the patients with a lipid-lowering therapy more than 3 months according to sex and age (right). LDL-C target values were determined in each patient according to the guidline (STEP 2) [1]. The first step (STEP 1) is mandatory and includes general recommendations (such as smoking cessation and lifestyle optimization), together with specified LDL-C target values for guiding lipid-lowering therapy [1]. The second step (STEP 2) outlines a more stringent regimen with intensified risk factor management und should be taken into consideration especially in patients with high-risk profiles; its application, however, depends on various factors, including 10-year CVD risk, comorbidities, frailty status, and particularly balancing lifetime risk and treatment benefit profile [1].

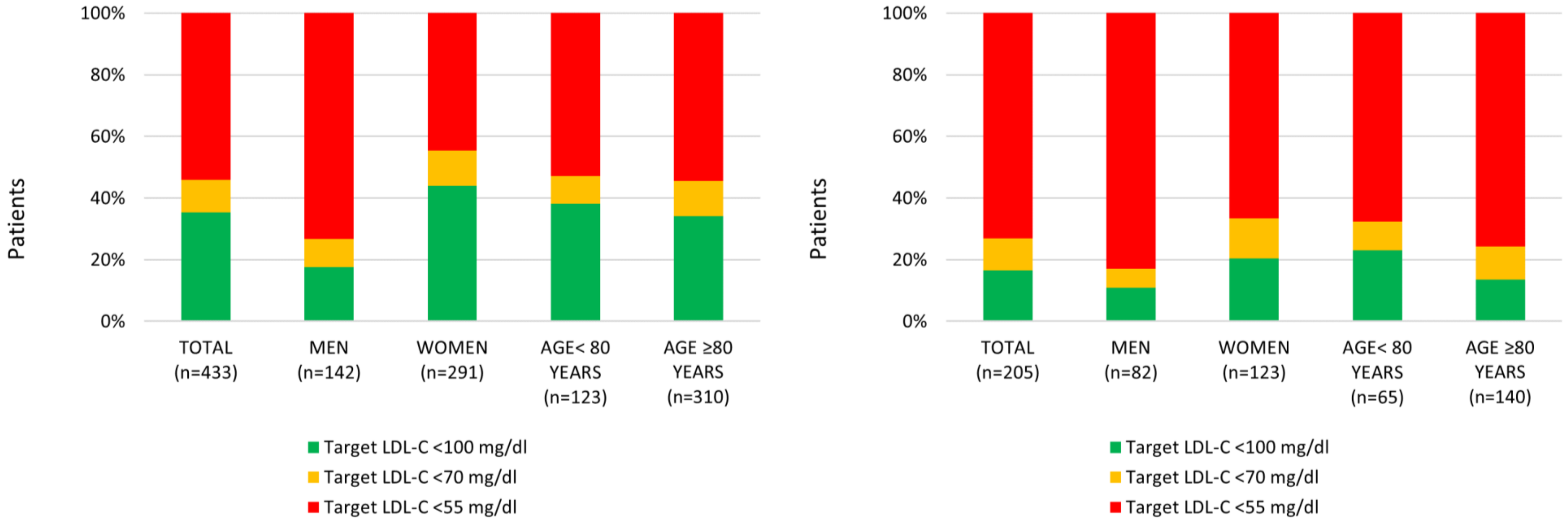

LDL-C = Low-Density-Lipoprotein Cholesterol

Literature

1. Visseren FLJ, Mach F, Smulders YM, Carballo D, Koskinas KC, Bäck M, Benetos A, Biffi A, Boavida JM, Capodanno D, Cosyns B, Crawford C, Davos CH, Desormais I, Angelantonio ED, Franco OH, Halvorsen S, Richard Hobbs FD, Hollander M, Jankowska EA, Michal M, Sacco S, Sattar N, Tokgozoglu L, Tonstad S, Tsioufis KP, van Dis I, van Gelder IC, Wanner C, Williams B; ESC Scientific Document Group. 2021 ESC Guidelines on cardiovascular disease prevention in clinical practice: Developed by the Task Force for cardiovascular disease prevention in clinical practice with representatives of the European Society of Cardiology and 12 medical societies With the special contribution of the European Association of Preventive Cardiology (EAPC). Rev Esp Cardiol (Engl Ed). 2022 May;75(5):429. English, Spanish. doi: 10.1016/j.rec.2022.04.003. PMID: 35525570.
